# Successful treatment of liver metastases arising from early gastric cancer achieved clinical complete response by nivolumab

**DOI:** 10.1186/s40792-018-0479-3

**Published:** 2018-07-05

**Authors:** Tsutomu Namikawa, Nobuko Ishida, Sachi Tsuda, Kazune Fujisawa, Eri Munekage, Jun Iwabu, Masaya Munekage, Sunao Uemura, Shigehiro Tsujii, Hiromichi Maeda, Hiroyuki Kitagawa, Michiya Kobayashi, Kazuhiro Hanazaki

**Affiliations:** 1 Department of Surgery, Kochi Medical School, Kohasu, Oko-cho, Nankoku, Kochi 783-8505 Japan; 2 Cancer Treatment Center, Kochi Medical School Hospital, Nankoku, Kochi Japan; 3 Department of Human Health and Medical Sciences, Kochi Medical School, Nankoku, Kochi Japan

**Keywords:** Gastric cancer, Nivolumab, Complete response, Chemotherapy, Biomarker

## Abstract

**Background:**

Although a recent randomized clinical trial has demonstrated that the objective response rate to nivolumab for metastatic gastric cancer was 11.2%, there was no patients confirmed complete response. Herein, we report on a case of liver metastasis arising from early gastric cancer in which a complete clinical response was achieved to nivolumab as third-line therapy.

**Case presentation:**

A 77-year-old man was referred to Kochi Medical School Hospital for the treatment of liver metastases from gastric cancer. The patient had undergone laparoscopic total gastrectomy with regional lymph node dissection 30 months prior for early gastric cancer, with a final diagnosis of T1N0M0, stage IA. The patient developed solitary splenic metastasis measuring 42 mm 28 months later and underwent splenectomy because there was no evidence of further metastatic lesions in any other organ. The patient was treated with S-1 plus oxaliplatin based on negative immunohistochemical staining of the resected specimens for human epidermal growth factor receptor 2 (HER2). Four months after the splenectomy, the patient developed multiple liver metastases and was treated with ramucirumab plus paclitaxel. Because of disease progression, the patient was administered 3 mg/kg, i.v., nivolumab every 2 weeks. After 4 cycles of systemic treatment using nivolumab, abdominal computed tomography revealed marked shrinkage of the liver metastases. After 12 cycles of nivolumab, the liver metastases had disappeared completely. The patient did not develop any adverse reactions, including immune-reactive adverse events, during treatment. The patient continues to receive nivolumab, and there is no evidence of disease recurrence in the 8-month period since starting nivolumab.

**Conclusions:**

To the best of our knowledge, this is the first case report in the English literature of a gastric cancer patient achieving a complete clinical response to nivolumab, and highlights the potential for successful treatment of metastatic gastric cancer using nivolumab.

## Background

Gastric cancer is a common malignancy; it is the seventh leading cause of cancer mortality worldwide, and the second most frequent cause of cancer-related deaths in Japan [1]. In metastatic or recurrent gastric cancer, first-line chemotherapy is recommended as the standard therapeutic regimen to prolong progression-free as well as overall survival. Platinum- and fluoropyrimidine-based combination therapies have been established worldwide as the first-line treatment regimens for advanced gastric cancer [2]. The standard regimens used as second-line treatments for gastric cancer are based on ramucirumab, which targets vascular endothelial growth factor receptor 2 (VEGFR2), with or without paclitaxel [3, 4]. Irinotecan monotherapy is recommended under some condition as second-line or as third-line treatment. Among these candidates, ramucirumab plus paclitaxel combination therapy is defined as a more recommended regimen used as second-line treatment for gastric cancer [3].

Immune checkpoint inhibitors are recently developed drugs that are being used for the treatment of malignant tumors, such as malignant melanoma, non-small cell lung cancer, and head and neck cancer [5]. A recent randomized phase 3 trial demonstrated that nivolumab, a fully human IgG4 monoclonal antibody inhibitor of the programmed death-1 (PD-1) receptor, had survival benefits in patients with metastatic advanced gastric cancer that was refractory to, or in patients who were intolerant of, standard therapy including two or more previous chemotherapy regimens [6].

Herein, we report on a case of liver metastases arising from early gastric cancer in which a complete clinical response was achieved to nivolumab as third-line therapy.

## Case presentation

A 77-year-old Japanese man was referred to Kochi Medical School Hospital for the treatment of liver metastases from gastric cancer. The patient’s past medical history revealed that he had undergone laparoscopic total gastrectomy with D1+ regional lymph node dissection, according to Japanese gastric cancer treatment guidelines 30 months prior for early gastric cancer [7]. The primary gastric cancer located in the upper third of the stomach, measuring 2.2 cm. The final diagnosis was T1N0M0, stage IA according to the 8th International Union Against Cancer (UICC) TNM classification [8], and the histological findings showed a well-differentiated adenocarcinoma coexisting with a solid-type poorly differentiated adenocarcinoma that had invaded the submucosal layer to a depth of > 2 mm. There was no lymph node metastasis in 35 dissected lymph nodes, no lymphovenous invasion. Twenty-eight months after the initial operation, abdominal computed tomography (CT) revealed a well-defined mass measuring 4.2 cm in diameter in the spleen, and ^18^F-2-deoxy-2-fluoro-glucose (FDG) positron emission tomography combined with CT imaging showed intense FDG uptake in the splenic mass, with no evidence of further metastatic lesions in any other organ. Under the clinical diagnosis of a solitary splenic metastasis, the patient underwent open splenectomy.

Histological examination confirmed the diagnosis of a solid-type poorly differentiated adenocarcinoma originating from the previous gastric cancer, and immunohistochemical analysis of the tumor showed no reactivity for human epidermal growth factor receptor 2 (HER2). Therefore, the patient was treated with chemotherapy using S-1 plus oxaliplatin. S-1 was given orally twice daily for the first 2 weeks of a 3-week cycle, at a dosage of 100 mg/day, and the patient received 100 mg/m^2^ of intravenous oxaliplatin on day 1 of each cycle. However, abdominal CT and magnetic resonance images showed multiple liver metastases 4 months after splenectomy, and was treated with ramucirumab plus paclitaxel as second-line treatment. The patients received ramucirumab 8 mg/kg intravenously on days 1 and 15, plus paclitaxel 80 mg/m^2^ intravenously on days 1, 8, and 15 of a 28-day cycle. After two courses of systemic treatment, abdominal contrast-enhanced CT revealed progression of the liver metastases.

The patient’s laboratory results, including serum carcinoembryonic antigen and cancer antigen 19–9, were within normal limits. Laboratory findings for markers of the systemic inflammatory response revealed normal total protein (6.6 g/dL; normal range, 6.6–8.1 g/dL), normal white blood cell (6.5 × 10^3^ mm^3^; normal range, 3.3–8.6 × 10^3^/mm^3^), neutrophil (3.8 × 10^4^/mm^3^; normal range, 1.6–5.9 × 10^4^/mm^3^), and lymphocyte (1.8 × 10^4^/mm^3^; normal range, 1.1–3.3 × 10^4^/mm^3^) counts, and slightly elevated C-reactive protein levels (0.7 mg/dL; normal range, < 0.14 mg/dL). Based on these findings, the Glasgow prognostic score (GPS) and peripheral neutrophil to lymphocyte ratio (NLR) were determined to be 0 and 2.1, respectively.

**Fig. 1:**
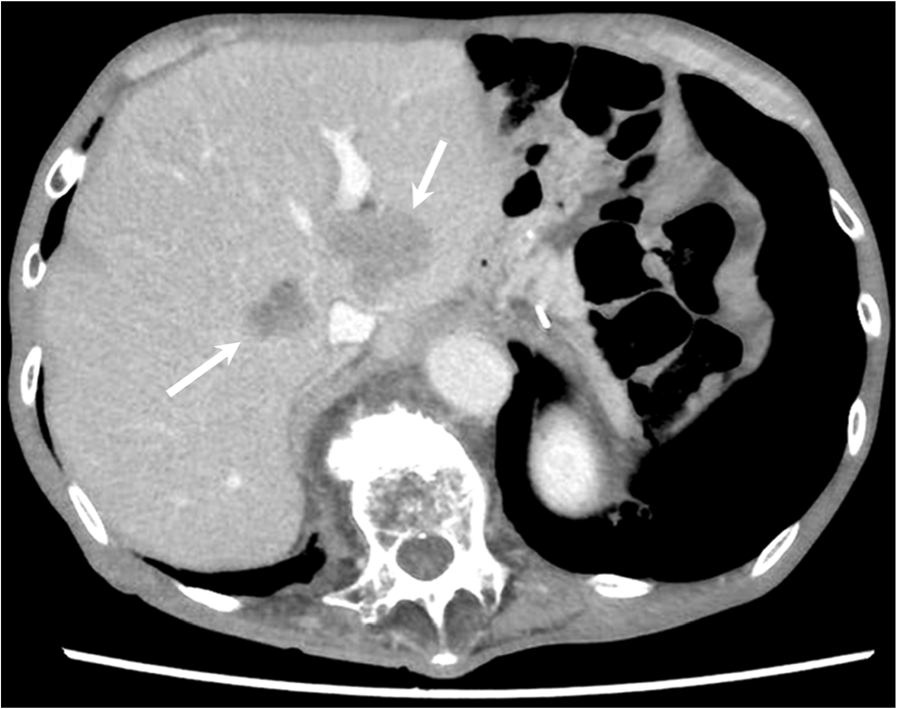
Initial findings on abdominal computed tomography (CT). Abdominal enhanced-contrast CT revealed multiple liver metastases in the bilateral lobe of the liver (arrows)

**Fig. 2:**
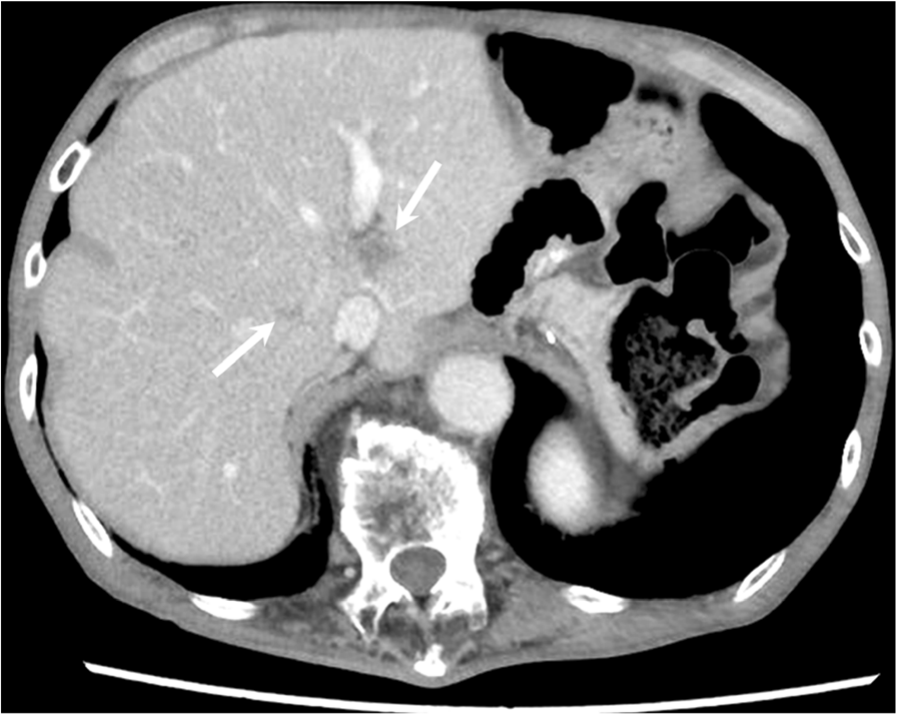
Abdominal computed tomography (CT) findings after four cycles of nivolumab. Abdominal enhanced-contrast CT revealed a marked reduction (by 55%) in liver metastases (arrows)

**Fig. 3:**
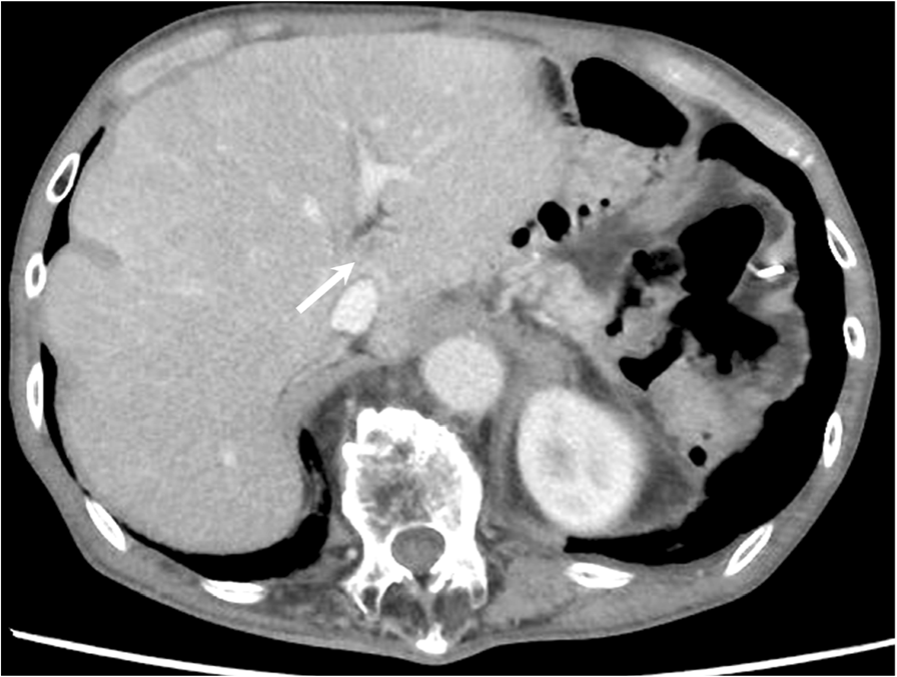
Abdominal computed tomography (CT) findings after eight cycles of nivolumab. Abdominal enhanced-contrast CT revealed a further reduction (by 82.6%) in liver metastases (arrow)

**Fig. 4:**
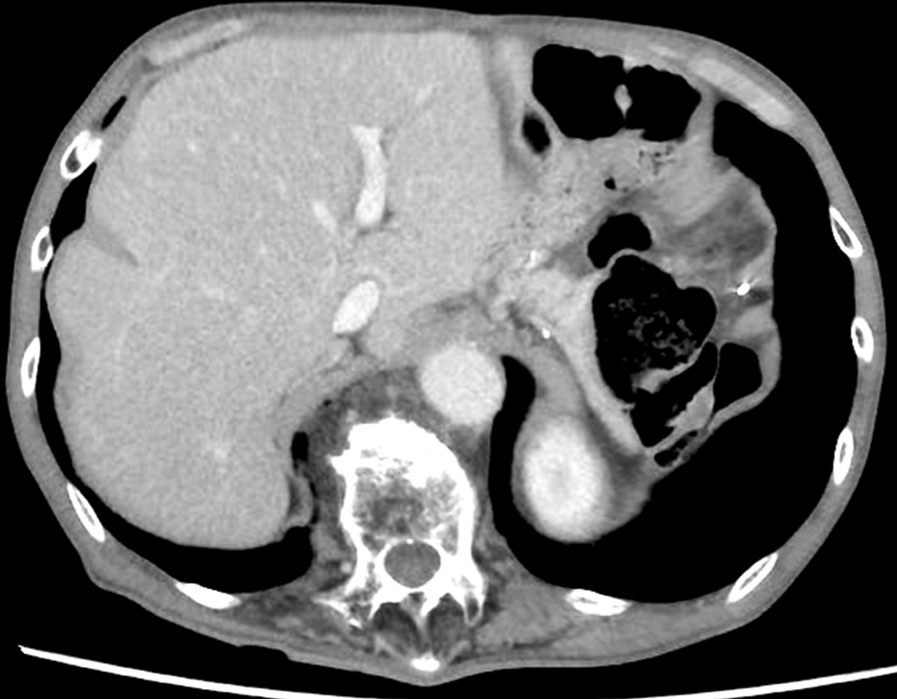
Abdominal computed tomography (CT) findings after 12 cycles of nivolumab. No liver mass lesions are seen on abdominal enhanced-contrast CT

Abdominal contrast-enhanced CT showed multiple well-defined mass lesions located in the bilateral lobe of the liver (Fig. 1). As third-line treatment for recurrent gastric cancer, the patient was administered 3 mg/kg, i.v., nivolumab every 2 weeks. After four cycles of systemic treatment with nivolumab, abdominal CT revealed a marked shrinkage of liver metastases, which indicated a partial response according to the Response Evaluation Criteria in Solid Tumors (RECIST 1.1) [9], with a 55.0% decrease in liver target lesions compared with baseline (Fig. 2). After eight cycles of nivolumab, abdominal CT revealed 82.6% decrease in liver target lesions (Fig. 3). After 12 cycles of nivolumab, abdominal CT revealed that all target lesions had disappeared; thus, a complete clinical response was achieved (Fig. 4). The patient did not develop any adverse reactions, including immune-reactive adverse events, during the course of treatment. The patient continues to receive nivolumab treatment, and there is no evidence of disease recurrence in the 8-month period since starting nivolumab.

## Discussion

In this case report, we present a rare case of liver metastases following curative resection of early gastric cancer in which a complete clinical response was achieved to nivolumab as third-line therapy. Although a recent randomized double-blind placebo-controlled phase 3 trial has demonstrated that the objective response rate to nivolumab for metastatic gastric cancer was 11.2%, there were no patients that confirmed complete response [6]. To the best of our knowledge, the present case report is the first case reported in the English literature of a gastric cancer patient achieving a complete clinical response to nivolumab therapy.

Although the liver is a common metastatic site of gastric cancer, the treatment for liver metastasis of gastric cancer has not been well established. According to Japanese gastric cancer treatment guidelines [7], chemotherapy is indicated for patients with unresectable or recurrent gastric cancer, including liver metastases. Despite the generally excellent outcome after curative surgery in patients with early gastric cancer, cancer recurrence is a rare event that can occur even after curative gastrectomy, with an incidence of 1.4–2.7% [10–12].

In previous randomized clinical trials, the rates of complete responses, as defined by RECIST, to drug treatment using chemotherapy and molecular targeted therapy were reported as 0.7–5.4% for first-line therapy [13, 14], 0.4–0.6% for second-line therapy [3, 4], and 0% for third-line therapy [6]. The objective response rate was lower for later than first-line therapy. The therapeutic efficacy of treatment regimens may have declined in patients who had undergone prior treatments as a result of decreased physical strength due to disease progression and/or cumulative cytotoxicity of the cytotoxic agents administered.

Recent studies have demonstrated that systemic inflammatory response markers, including GPS and NLR, are associated with prognosis in cancer patients [15, 16]. Previous investigators demonstrated that the unresectable advanced gastric cancer patients with high NLR were significantly associated with worse overall survival when the cut-off values were set at 3.0–4.0 [16–19]. In the present case, NLR was low and GPS was 0, which indicate maintained host immune responses to the tumor. Neutrophilia is an inflammatory response that inhibits the immune system by suppressing the cytolytic activity of immune cells, whereas lymphopenia is a surrogate for impaired cell-mediated immunity. NLR, calculated as neutrophil counts/lymphocyte counts, has been suggested as a marker for the general immune response to various stress stimuli [16, 20]. Furthermore, systemic inflammatory responses can indicate nutritional decline, which could contribute to tumor progression [21].

Ameratunga et al. reported shorter overall survival in patients with advanced solid tumors using an NLR cut-off value of 5.0 [22]. Similarly, Nakaya et al. reported that progression-free survival was worse in advanced non-small-cell lung cancer patients with a high NLR when the cut-off value was set at 3.0 [23], Furthermore, meta-analysis to investigate the prognostic utility of baseline NLR in patients receiving immune checkpoint inhibitors showed that a high NLR was associated with poorer outcomes [24]. Therefore, the host inflammatory response markers including NLR may be important not only in the development and progression of cancer but also in predicting responses to immune checkpoint inhibitors.

Immune checkpoint-targeted therapy has emerged as a promising treatment strategy with considerable benefits in many cancer types; however, it is not suitable for all patients [5, 6]. Although the expression of PD-1, and its ligands PD-L1 and PD-L2, or microsatellite instability (MSI) profiles are frequently used to select patients for immunotherapy trials and appear to be correlated with treatment response, a universal biomarker has not been identified as yet that can accurately predict patients who are more likely to respond to immunotherapy [5, 25]. In order to improve the efficacy of immune checkpoint-targeted therapy, biomarkers that can predict patient responses to immunotherapy need to be developed [25, 26].

When a clinical complete response is achieved, the significance in the continuation of treatment is unknown. Cho et al. reported that three patients developed recurrence among 22 patients who achieved a pathological complete response with neoadjuvant chemotherapy [27]. Although a complete response induced by drug treatment is associated with the better prognosis of patients, the continuation of treatment seems to be necessary to aim long-term survival at this time.

## Conclusions

The response of the present patient to nivolumab indicates that it may prolong the survival of patients with metastatic or recurrent gastric cancer. In addition, nivolumab exhibited good safety and tolerability profiles in the present case, indicating its potential use for the successful treatment of metastatic gastric cancer. In addition to the accumulation of additional cases, further investigations, including large-scale, global, multicenter clinical studies, are needed to determine whether nivolumab is suitable for use in first- or second-line therapy, and whether it should be administered alone or in combination with other therapies.

## Abbreviations

VEGFR2: Vascular endothelial growth factor receptor 2
PD-1: Programmed death-1
HER2: Human epidermal growth factor receptor 2
CT: Computed tomography
GPS: Glasgow prognostic score
NLR: Neutrophil to lymphocyte ratio
RECIST: Response evaluation criteria in solid tumors
MSI: Microsatellite instability
